# Social exclusion and mental health among older adults: cross-sectional evidence from a population-based survey in India

**DOI:** 10.1186/s12888-022-04064-1

**Published:** 2022-06-18

**Authors:** Babul Hossain, Varsha P. Nagargoje, Md Illias Kanchan Sk, Jyoti Das

**Affiliations:** International Institute for Population Sciences, Mumbai, 400088 India

**Keywords:** Social exclusion, Depressive symptoms, Older adults, India

## Abstract

**Background:**

Social exclusion has far-reaching consequences that extend beyond regular activities and access to resources and knowledge; social exclusion is a major social determinant of health. However, there is a lack of evidence on social exclusion and health outcomes among India’s older adults. Thus, the current study investigates the association of social exclusion with depressive symptoms among Indian older adults.

**Methods:**

This study used information on 30,366 older adults from Longitudinal Ageing Study in India (LASI) wave-1, 2017-2018. Social exclusion scores were calculated, and two broad domains of social exclusion, i.e., exclusion from civic activity & social relations and exclusion from services, were considered in the study. The depressive symptom was calculated using the CES-D score. Using logistic regression models, the average marginal effects of selected covariates and domains of social exclusion on depressive symptoms were estimated to assess the links between social exclusion and depressive symptoms.

**Results:**

With the increase in the social exclusion score in the selected domains, the prevalence of depressive symptoms among older also increased. Elderly persons who do not vote or live alone in the domain of being excluded from civic & social activities and older adults excluded from services were observed to have a higher prevalence of depressive symptoms. Adjusting for sociodemographic factors, the average marginal effects suggested that older with four scores of civic activity & social relation exclusion, two scores of service exclusion and four scores of overall social exclusion were estimated to have a higher prevalence of depressive symptoms, respectively.

**Conclusions:**

This study’s findings shed light on social exclusion and its relationship to depressive symptoms among older Indians. Older health care services should be expanded in breadth while also addressing social exclusion, resulting in considerable improvements in older individuals’ mental health.

**Supplementary Information:**

The online version contains supplementary material available at 10.1186/s12888-022-04064-1.

## Background

The phrase “social exclusion” refers to the process through which people and groups become separated from social relationships and institutions [1, 2]. The Report of World Social Situation (2016) by United Nations, also used the phrase “social exclusion” to represent people’s lack of involvement in, or exclusion from, economic, political, cultural, civic, and/or social life [3]. WHO also defined social exclusion as it comprises of dynamic, multifaceted processes driven by unequal power relationships interacting across four key aspects economic, political, social, and cultural at many levels, such as individual, home, group, community, national, and global [4]. The term social exclusion has also been simultaneously used as social isolation as the absence of social cohesion [5]. Social exclusion is considered as a fundamental problem as the dimensions extend to poverty and low income, unemployment, poor educational attainment, lack of participation in political, civic, and cultural activities, the disintegration of family and the discrimination occurs based on caste, creed, gender and age [6–8]. Thus, individuals and groups left out of these processes have limited influence over the attitudes, norms, institutions, and policies that lead to social exclusion in the first place [9, 10]. Correspondingly, it is also probable that the United Nations adopted the concept of social exclusion to explain its link with low quality of life and poor health condition [4]. Numerous aspects of social exclusion have been identified, and research has identified five major domains of social exclusion: material and financial resources, including income and material security; civic activities, including political decision-making; social relations, including meaningful relationships with friends and family; essential services in terms of social and health care, access to information, and neighbourhood cooperation [11, 12].

The implications of social exclusion are widespread in individual or group lifestyles. It is also important to mention that social exclusion is associated with worsening physical and mental health outcomes. Although, previous literature indicated that there might be a bidirectional relationship exist between social exclusion and health. For example, studies have reported that poor health is an important predictor of social exclusion [9, 13]. On the other hand, existing evidence also suggests that social exclusion can contribute to poor health and potentially exacerbate physical disease, lower physical activity, and mental health issues [14–16]. A European population-based cross-sectional study found that exclusion from material resources and basic services can negatively affect general health and life satisfaction [17]. While according to Sacker and colleagues, socially excluded people were more likely to report long-term illness/disability and had worse scores on a general health index and self-rated health [18]. Holt-Lunstad’s study also mentioned that social connection is a key protective factor, while social isolation is a risk factor for to catastrophic long-term repercussions, including premature death [19]. Study findings of 14 European countries demonstrated that higher levels of financial and social exclusion were related to poorer quality of life, concluding that the material exclusion was strongly associated with worsening the quality of life [20]. Social exclusion is not just related to poor physical health status [21, 22] but also has a negative link with the mental health of an individual [23, 24].

While Baumeister and Tice’s (1990) social exclusion theory proposed that the primary source of anxiety or depression among individuals is the result of their perceived exclusion from social groups [25]. Although according to Østbye et al. (2000), social exclusion and lower perceived health may be a consequence rather than a cause of the depressive symptoms because dysphoric individuals, particularly older adults, tend to participate less likely in social activities [26]. Similarly, Kummitha (2015) also stated that social exclusion was associated with poor mental health [27].

Saying so, most countries are experiencing a demographic transition with a proportional increase in the older population. Though countries are striving towards universally accessible health care benefits for all their people, older adults, in particular experiencing systematic exclusion, remain vulnerable to worst health outcomes [28]. Compared to the younger age cohort, older persons have higher rates of ailments, disability, and partial or complete financial reliance on others. Age-based discrimination (ageism), making the older population more prone to social exclusion in communities [11, 29–31]. Looking at the increased vulnerability, inequities and hardship among the aging individuals, social exclusion among older adults is gaining more attention in the present time. Also, in the wake of emerging global challenges, social exclusion becomes a chief concern for older adults’ mental health [29].

In this context, several studies are evident that later-life social exclusion is significantly associated with increased mental health-related issues. Socially excluded older individuals lack emotional and concrete support, which affects their mental health and is also responsible for deteriorating their quality of life. For Instance, according to the findings of a Chinese study, social exclusion indicators were more relevant than other socio-demographic factors in predicting higher levels of depression in older persons [32]. Furthermore, a study revealed that decreasing participation in community activities might increase the burden of depression among the old-age population [33]. While looking at the gender dimension of social exclusion and mental health, it was found that women were more vulnerable to exclusion, especially in terms of housing, education, and health, which causes depressive symptoms in women. In contrast, men were more vulnerable than women in terms of social participation exclusion leading to higher depression [34, 35]. According to one study, there was a significant link between social exclusion and cognitive impairment in China, particularly among rural old-age women who were most vulnerable to social exclusion and cognitive impairment [36]. Rahman and Singh (2018) have shown that disability among older adults leads to lower social cohesion in aged people [37]. Few studies also pointed out that being older female, widowed, unemployed, urban residents, belonging to lower classes, living alone, and not having formal education makes older people more susceptible to severe social exclusion that may also alter their health status [30, 37].

India is now undergoing a significant demographic change, with reduced infant and adult mortality and longer life expectancy, resulting in substantial growth in the proportion of old-age people. According to the 2011 census of India, the Indian older population (i.e., aged 60 years and over) accounts for 8.6% of the total population [38]. By 2050, this percentage will be about 19% [39, 40]. Although there are studies in Indian context focused on social exclusion and depression in older persons separately, the research is limited. For instance, In a multi-country analysis, including India, Rahman and Singh (2018) examined the link between disability and social cohesion among older adults [37]. The study results demonstrated that impairment has a strong and significant impact on the social cohesiveness of older individuals [37]. Hossain et al. (2021) have explored the association between physical limitations and self-reported depressive symptoms among Indian older adults considering marital status as a moderator in such association [41]. Other studies by Chauhan et al. (2021) and Srivastava et al. (2021) have also evaluated the repercussions of living arrangements and other predictor variables on depression among older adults in India [42, 43].

With most older adults relying on other family or household members for their day-to-day activities, economic needs, and emotional support in late life, understanding the social exclusion of older adults and its role in mental health has become crucial for India. Based on the reviewed literature, it is clear that many empirical studies worldwide have highlighted the range of aspects of social exclusion and its association with older adults’ mental health or well-being. So far, none of the researchers has considered the role of social exclusion in altering the mental health of Indian old-age people. There is still a shortage of investigation about the influences of social exclusion on the mental health of the older adults in Indian settings. Hence, it raises concerns about understanding how social exclusion could play a role in understanding and analysing the depression persisting among the Indian older population. Thus, this study will focus on the association between social exclusion and depressive symptoms of older adults in India.

## Material and methods

### Data source

The dataset used in the present study has been sourced from the Longitudinal Ageing Study in India (LASI) wave 1, 2017-2018, a national and state representative longitudinal large-score survey on ageing and health, especially for older adults aged 45 and above [40]. LASI provides accurate, reliable, and continuous scientific data on social and economic well-being, health such as physical and mental status, and health care utlisation. The selected sample comprises non-institutionalized Indian residents sorted through the multistage stratified probability cluster sampling design from all 30 states (excluding Sikkim) and 6 Union Territories of India [40].

LASI adopted a four-stage sampling design in urban areas and a three-stage sampling design in rural areas. At the initial stage, the list of sub-districts (Tehsils/Talukas) was considered as Primary Sampling Units (PSUs) for each state/UT based on the 2011 Indian census. Then the PSUs in each region were selected using Probability Proportional to Size (PPS) sampling with a number of households in each PSU as the size measure. The next step involved selecting a fixed number of Secondary Sampling Units (SSUs), wards from urban areas and villages from rural areas of the selected PSUs. Finally, a fixed number of rural households were chosen from selected villages using systematic sampling at the third stage. However, sampling in urban areas required one more stage. In the third stage, one Census Enumeration Block (CEB) was randomly selected from each selected urban ward. Finally, a fixed number of households from this CEB were systematically selected in the fourth stage. More detailed information about the sampling framework and selection of sample size is available in the national report of LASI, wave 1, 2017-18, India [40].

### Study sample

**Fig. 1:**
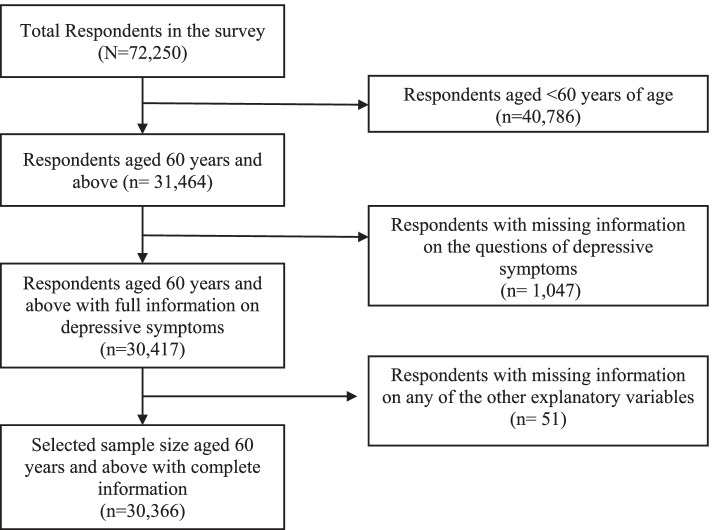
Flow chart for the sample selection for the study analysis

LASI wave one provided information on the total sample of 72,250 aged 18 and above without any missing value in age reporting. The present study population was older adults aged 60 years and above. Thus below 60 years old (*n* = 40,786), samples were dropped. The dependent variable was depressive symptoms among the older adults. Further, 1047 respondents were with missing information on the questions of depressive symptoms were dropped from the sample [42]. Also, information on any other explanatory variables, such as marital status, educational status, etc., consists of missing values that we have dropped (*n* = 51). Hence, after dropping the sample containing missing values, the final sample became 30,366. Figure 1 provides a flowchart of the final sample selection for the study.

### Variable description

#### Outcome variable

##### Mental health- depressive symptoms

In the present study depressive symptom was considered for mental health status. Radloff (1977) initially developed a screening tool, a short self-report score comprising 20 items to calculate the depressive symptoms [44]. However, the present study had adopted the shortened version of the Center for Epidemiological Studies- Depression (CES-D) score developed by Anderson et al. (1994), also used in the LASI for the measurement of depressive symptoms [45]. The CES-D by Anderson (1994) comprised seven negative symptoms, i.e., fear of something, low energy, trouble concentrating, feeling alone, feeling depressed, bothered by things, and everything is an effort, while three positive symptoms included feeling happy, hopeful, and satisfied [45]. For all these ten symptoms, individuals had responded rarely or never, i.e., < 1 day, sometimes, i.e., 1 or 2 days, often, i.e., 3 or 4 days, and most or all of the time, i.e., 5-7 days in a week prior to the interview in the LASI. For the negative symptoms, rarely or never and sometimes were scored zero while often and most or all of the time categories were scored one. At the same time, scoring was reversed for three positive symptoms. The overall score varies from zero to 10, and the score of four or more was considered to calculate the prevalence of depressive symptoms [40].

#### Explanatory variable

##### Social exclusion

Two domains of social exclusion were considered: civic activity-social relations and services.

##### Exclusion from civic activity & social relation

The civic activity and social activities had been used as a separate domain to define social exclusion in existing literature [27, 46, 47]. However, due to data restrain we have amalgamated voting behaviour with other variables of social relation and created exclusion from civic activity & social relation. The measure of exclusion from the civic activity and social relations was derived from a series of civil activity and social relations questions. For the civic activity question, whether the respondent voted in the last panchayat/municipal/assembly/parliament elections (no/yes) were considered. While for the social activity questions on attended political/community/organization group meetings, cultural performances /shows/Cinema, religious functions /events such as bhajan/satsang/prayer (daily, several times a week, once a week, several times a month, at least once a month rarely/Once in a year) and living alone were considered. For attending political/community/organization group meetings, cultural performances /shows/Cinema and religious functions /events, 0 were coded for no if individuals responded never/not relevant and 1 for yes other than never/not relevant categories. While non-voter behaviour was categorized as 0 if the individual responded not casting a vote in the last panchayat/municipal/assembly/parliament elections and 1 if the individual did [27]. Simple count approach was used to calculate the score. Thus, the selected items were summed. Respondents’ total score for the civic activity & social relation exclusion range from 0 to 4, where a higher score indicated more exclusion from civic activity & social relation of the elderly.

##### Exclusion from service

Participants had asked whether in the past they received poorer service than other people at restaurants/stores or poorer treatment than other people from doctors or hospitals where participants’ response was recorded in following categories: almost every day, at least once a week, a few times a month, a few times a year, less than once a year and never [29, 48]. The participants’ responded never was categorised as no coded 0; otherwise, yes coded as 1. Similar to exclusion from civic activity & social relation, the items were summed and respondents’ total score for the service exclusion score range from 0 to 2, where higher score indicated more exclusion from services.

##### Overall social exclusion

For the overall social exclusion score, all the items to define the domains of social exclusion were summed where the total score range from 0 to 6. Higher score indicated the higher social exclusion of the individual.

### Covariates

Other covariates considered and controlled in the analysis age (60-69, 70-79 and 80+), place of residence (rural and urban), gender (men and women), marital status (married and non-married), educational status (no formal education and formal education), wealth index (poor, middle and rich).

### Statistical analysis

The present study used a bivariate technique to estimate the prevalence of depressive symptoms by sociodemographic variables and domains of social exclusion. Sampling weights provided by the LASI were employed. Further, the logistic regression technique was applied to estimate unadjusted and adjusted average marginal effects of selected domains of social exclusion on the probability of depressive symptoms. Each marginal effect was averaged over the sample used in the respective regression. The results were presented in an percentage points (pp) with a 95% confidence interval (CI). All the statistical analysis had done using STATA (version 16) and MS excel program.

## Results

Respondents’ socio-demographic characteristics are summarised in Table 1. Around 60% of the older adults were in the 60 to 69 years age group. The Majority of the samples were from a rural area (71%). Nearly 62% of the samples were married. More than half of the sample had no formal education (57%). Most of the elderly belonged to the poorer wealth index (43%).

**Table 1:** Sample characteristics, LASI wave 1, 2017-18 (*N* = 30,366)

Characteristics	N	%
**Age group**		
60-69	18,062	59.5
70-79	9039	29.8
80+	3265	10.8
**Gender**		
Men	14,315	47.1
Women	16,051	52.9
**Place of residence**		
Rural	21,581	71.1
Urban	8785	28.9
**Marital status**		
Married	18,752	61.8
Non-married	11,614	38.3
**Educational status**		
No formal education	17,144	56.5
Formal education	13,222	43.5
**Wealth index**		
Poor	13,148	43.3
Middle	6376	21.0
Rich	10,842	35.7

Note: LASI provided sampling weights were applied

Responses to the items included in the domains of social exclusion are presented in Table 2. In the exclusion from civic activity & social relation domain, nearly 4% of the individuals were non-voter. More than three-fourths of the sample did not attend social activities such as political/community/organization group meetings (86%) and cultural performances /shows/Cinema (75%). Almost half of the older adults (47%) did not attend religious functions /events such as bhajan/satsang/prayer. At the same time, approximately 6% of older adults live alone. In exclusion from services, 7% of the elderly sample reported receiving poorer services than other people at stores/restaurants, and 6% reported receiving poorer services than other people from a doctor.

**Table 2:** Participants’ response to different social exclusion indicators (*N* = 30,366)

Social exclusion domain	N	%
***Exclusion from Civic activity & social relation***		
**Non-voter behaviour**		
Non voter	1087	3.6
Voter	29,279	96.4
**Attended political/ community/ organization group meetings**		
No	26,176	86.2
Yes	4190	13.8
**Attended cultural performances/ shows/ cinema**		
No	22,724	74.9
Yes	7642	25.1
**Attended religious functions/ events such as bhajan/ satsang/ prayer**		
No	14,291	47.1
Yes	16,075	52.9
**Living alone**		
No	28,628	94.3
Yes	1738	5.7
***Exclusion from services***		
**Received poorer services than other people at store/ restaurants**		
No	28,305	93.2
Yes	2061	6.8
**Received poorer services than other people from doctor**		
No	28,491	93.8
Yes	1875	6.2

Note: LASI provided sampling weights were applied

Table 3 represents the prevalence of depressive symptoms among the older adults using the CES-D score. Almost 30% of older adults had depressive symptoms. With the increase in age, the prevalence of depressive symptoms also increased. Almost one-third (31%) of the rural-dwelling older adults reported depressive symptoms, while 27% of the urban-dwelling older adults reported depressive symptoms. Women (33%) had a higher prevalence of depressive symptoms than men (27%). While non-married (35%) and individuals with no formal education (34%) reported higher prevalence than their counterparts. Also, older adults with the rich wealth index (42%) had a higher prevalence of depressive symptoms than other groups.

**Table 3:** Prevalence of depressive symptoms among older adults by socio-demographic characterises

Characteristics	N	%	***P***-value
**Level of Depressive symptoms**	30,366	30.3	
**Age group**			
60-69	18,062	28.5	0.000
70-79	9039	31.4	
80+	3265	36.2	
**Place of residence**			
Rural	21,581	31.3	0.000
Urban	8785	27.5	
**Gender**			
Men	14,315	27.4	0.000
Women	16,051	32.8	
**Marital status**			
Married	18,752	27.3	0.000
Non-married	11,614	34.8	
**Educational status**			
No formal education	17,144	34.3	0.000
Formal education	13,222	24.9	
**Wealth index**			0.000
Poor	13,148	38.6	
Middle	6376	37.8	
Rich	10,842	41.6	

Note: LASI provided sampling weights were applied

Table 4 depicts the prevalence of depressive symptoms by the items included in the domains of social exclusion. In the exclusion from civic activity & social relation domain, non-voter (42%) had a higher prevalence of depressive symptoms than voters (30%). At the same time, not much difference in the prevalence of depressive symptoms was observed for attending political/community/organization group meetings, cultural performances /shows/Cinema and religious functions /events. Although, elderly living alone (80%) had a significantly higher prevalence of depressive symptoms. While in the exclusion from service domain, the elderly received poorer services than other people at store/restaurants (56%) or from a doctor (58%) had a higher prevalence of depressive symptoms than their counterparts.

**Table 4:** Prevalence of depressive symptoms among older adults by various different social exclusion indicators

Social exclusion domain	N	%	P-value
***Exclusion from Civic activity & social relation***			
**Non-voter behaviour**			0.000
Non-voter	1087	41.7	
Voter	29,279	29.8	
**Attended political/ community/ organization group meetings**			0.088
No	26,176	30.4	
Yes	4190	29.3	
**Attended cultural performances/ shows/ cinema**			0.699
No	22,724	30.3	
Yes	7642	30.1	
**Attended religious functions/ events such as bhajan/ satsang/ prayer**			0.000
No	14,291	30.6	
Yes	16,075	29.9	
**Living alone**			0.000
No	28,628	21.5	
Yes	1738	79.8	
***Exclusion from service***			
**Received poorer services than other people at store/ restaurants**			0.000
No	28,305	28.2	
Yes	2061	55.9	
**Received poorer services than other people from doctor**			0.000
No	28,491	28.3	
Yes	1875	58.4	

Note: LASI provided sampling weights were applied

**Fig. 2:**
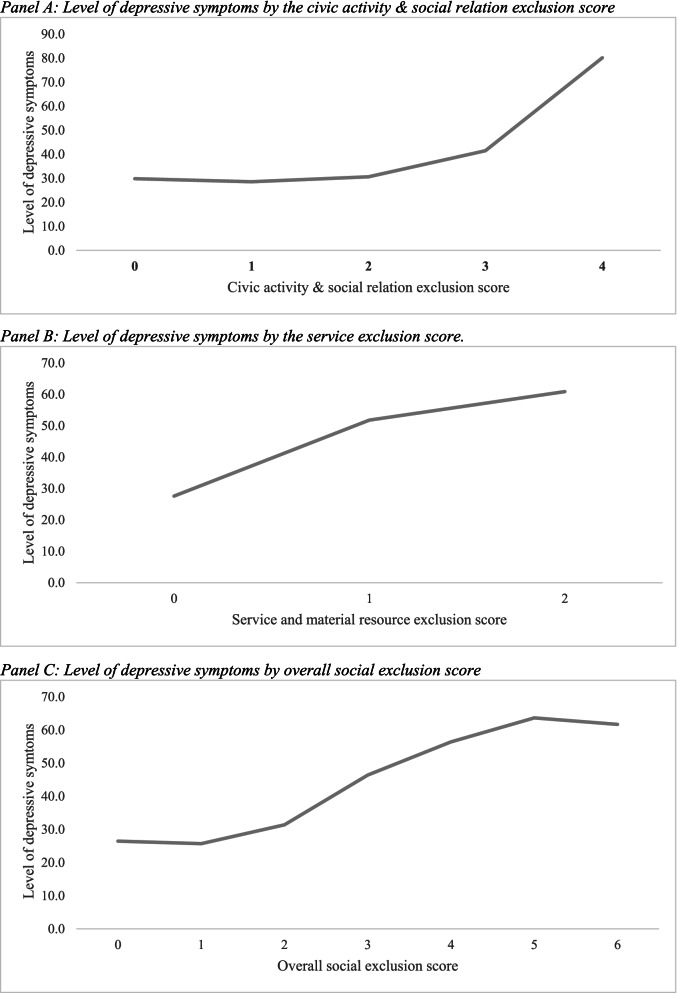
Level of depressive symptoms by the domains of social exclusion. Panel **A**: Level of depressive symptoms by the civic activity & social relation exclusion score. Panel **B**: Level of depressive symptoms by the service exclusion score. Panel **C**: Level of depressive symptoms by overall social exclusion score

Figure 2 shows the prevalence of depressive symptoms among the elderly by social exclusion scores in different domains. The level of depressive symptoms increased with the higher score of civic activity & social relation exclusion and service exclusion (Panel A and Panel B). A similar pattern also had been observed for the overall social exclusion score (Panel C).

Table 5 shows average marginal effects (AME) represented in percentage points (pp) from logistic regressions. We have used five models in the multivariate analysis. Model 1 indicates the adjusted average marginal effects of various sociodemographic characterises on depressive symptoms. Model 2 includes the unadjusted average marginal effects of selected domains of social exclusion on depressive symptoms. Further, Model 3, Model 4 and Model 5 shows the adjusted average marginal effects of civic activity & social relation exclusion score, service exclusion score and overall social exclusion score on depressive symptoms, respectively.

**Table 5:** Averaged marginal effects on the probability of depressive symptoms among older adults aged 60 years and above in India, LASI wave 1, 2017-18 (N = 30,366)

	Model 1	Model 2	Model 3	Model 4	Model 5
	AME in pp (CI at 95%)	AME in pp (CI at 95%)	AME in pp (CI at 95%)	AME in pp (CI at 95%)	AME in pp (CI at 95%)
**Social exclusion**					
***Civic activity & social relation exclusion score***					
0 (ref.)					
1		−0.021 (−0.035 -0.006)	−0.031 (−0.046 -0.017)		
2		0.022***(0.008 0.036)	− 0.001 (− 0.016 0.013)		
3		0.123***(0.094 0.153)	0.068***(0.039 0.097)		
4		0.333***(0.166 0.5)	0.263***(0.091 0.435)		
***Service exclusion score***					
0 (ref.)					
1		0.236***(0.21 0.262)		0.226***(0.2 0.252)	
2		0.351***(0.322 0.381)		0.346***(0.316 0.376)	
***Overall social exclusion score***					
0 (ref.)					
1		−0.007 (−0.021 0.007)			− 0.015* (− 0.03 -0.001)
2		0.06***(0.045 0.074)			0.041***(0.026 0.055)
3		0.212***(0.187 0.236)			0.175***(0.151 0.2)
4		0.326***(0.277 0.374)			0.291***(0.243 0.34)
5		0.282***(0.14 0.424)			0.228***(0.087 0.369)
6		0.428 (−0.105 0.962)			0.362 (−0.202 0.927)
**Age group**					
60-69 (ref.)					
70-79	0.024***(0.012 0.036)		0.023***(0.012 0.035)	0.025***(0.013 0.036)	0.022***(0.01 0.033)
80+	0.058***(0.04 0.076)		0.055***(0.037 0.073)	0.06***(0.042 0.077)	0.049***(0.032 0.067)
**Place of residence**					
Rural (ref.)					
Urban	−0.015**(−0.026 -0.003)		− 0.016***(− 0.027 -0.005)	−0.015***(− 0.026 -0.004)	−0.016***(− 0.027 -0.005)
**Gender**					
Men (ref.)					
Women	0.012**(0.001 0.024)		0.012**(0.001 0.024)	0.016***(0.005 0.028)	0.012**(0 0.023)
**Marital status**					
Married (ref.)					
Non-married	0.055***(0.043 0.067)		0.047***(0.035 0.059)	0.052***(0.04 0.064)	0.037***(0.025 0.049)
**Educational status**					
No formal education (ref.)					
Formal education	−0.045***(−0.056 -0.033)		−0.043***(− 0.054 -0.031)	−0.035***(− 0.046 -0.024)	−0.035***(− 0.046 -0.023)
**Wealth index**					
Poor (ref.)					
Middle	−0.034***(− 0.047 -0.02)		−0.034***(− 0.047 -0.02)	−0.033***(− 0.047 -0.02)	−0.033***(− 0.046 -0.019)
Rich	−0.024***(− 0.035 -0.012)		−0.025***(− 0.037 -0.014)	−0.025***(− 0.036 -0.014)	−0.026***(− 0.037 -0.014)

Note: AME denotes Averaged marginal effects; pp. denotes percentage points. Estimated averaged marginal effects on probability of the depressive symptoms from logistic regressions

The result from Model 1 showed that the prevalence of depressive symptoms was estimated to be 5.8 pp. (95% CI: 4.0, 7.6) higher in older adults aged 80 and above years than older adults aged 60 to 69 years. At the same time, urban-dwelling and formal educated older adults were estimated to have lower prevalence of depressive symptoms than their counterparts. Whereas women (pp = 1.2, 95% CI: 1.0, 2.4) and non-married older adults (pp = 5.5, 95% CI: 4.3, 6.7) estimated to have higher prevalence of depressive symptoms. After accounting for other characteristics, the wealth gradient in depressive symptoms remained significant, with a higher wealth index estimated to have a lower prevalence of depressive symptoms.

The unadjusted average marginal effects of selected domains of social exclusion on depressive symptoms indicates that prevalence of depressive symptoms was estimated to be higher with increased score of civic activity & social relation exclusion, service exclusion and overall social exclusion (Model 2). Older adults with four scores of civic activity & social relation exclusion was estimated to have 33.3 pp. (95% CI: 16.6, 50.0) higher prevalence of depressive symptoms compare to those older adults with zero score of civic activity & social relation exclusion. At the same time, among the older adults with score of two in exclusion from service (pp = 35.1, 95% CI: 32.2, 38.1) and score four in overall social exclusions (pp = 32.6, 95% CI: 27.7, 37.4) were estimated to have higher prevalence of depressive symptoms.

While controlling the sociodemographic characterises, the older adults with four scores of civic activity & social relation exclusion estimated to have 26.3 pp. (95% CI: 9.1, 43.5) higher and two scores of service exclusion had 34.6 pp. (95% CI: 31.6, 37.6) higher prevalence of depressive symptoms, respectively (Model 3 and 4). At the same time, older adults with four scores of overall social exclusions were estimated to have 29.1 pp. (95% CI: 24.3, 34.0) higher prevalence of depressive (Model 5).

Although the results in the Table 5 only includes the individuals with complete information on the questions on depressive symptoms, a similar additional analysis was carried out by including the individuals with missing information on the questions on depressive symptoms (See Appendix Table 1). Considering the missing value for the analysis, we found there were difference in the average marginal effects (AME) of selected domains of social exclusion on the depressive symptoms.

## Discussion

Using a nationwide representative sample, the current research intends to examine the relationships between social exclusion and health outcomes in terms of depression among older persons in India. As India undergoing rapid demographic transition as a result of rising life expectancy, proportionate growth in the population aged 60 years or older, and also the economic, social, and psychological changes associated with the ageing process, the findings indicate specific associations between various dimensions of social exclusion and depressive symptoms.

We find that depressive symptoms are more prevalent in those aged 80 and above, women, rural residents, unmarried, and without a formal education. Notably, older persons who are members of the wealthy household also have a higher prevalence of depressive symptoms. While focusing on the principal study objective; assessing social exclusion and depression symptoms link, our research highlights that the prevalence of depressive symptoms is higher among non-voters and older adults who live alone in the domain of exclusion from civic activity and social relationships, as well as among older adults who are excluded from service. While the regression analysis demonstrates that when the number of scores in specific domains of social exclusion and also overall social exclusion increases, depressive symptoms among older adults is likely to increase even after controlling demographic and socioeconomic characteristics.

Our findings indicate that older people who live alone are more likely to have depressive symptoms, which is consistent with prior studies. As it has been observed that older adults who live with their offspring or another family member get better social support, which has a direct effect on their materialistic needs and care related aspects [49, 50]. In contrary, excluded from social aspect of life for instance living alone not only deprives the elderly of necessities and supplies, but also leaves them feeling helpless, which may increase depression level in older individuals [51, 52]. Studies argued that the exclusion in the family level where living alone or single-person households is taken as a proxy were significant predictors of social exclusion and affecting older adults physical and mental health condition [46, 48]. Tong and colleagues conducted research on social exclusion and depression among the elderly in China and revealed that structural changes in the economic due to change in living arrangement and loneliness significantly correlate with increased depressive symptoms among older Chinese persons [32]. In Indian culture, older adults are traditionally cared for by their household members and children. Furthermore, older adults commanded a sense of authority, as they are often seen as the head of the household or as decision-making members of the household in India. On the other hand, active participation in religious activity and other community-level activities is common traits of ageing in India. By engaging in several social and community activities, older adults sense integrated. However, older individuals who live alone or who do not participate in religious and communal activities are not only more likely to experience loneliness, but they also lose these social aspects of daily life, which may exacerbate their sense of social exclusion and, consequently, increase their depressive symptoms. While we also identify that higher score of exclusion from civic participation and social relationships are strongly linked with a higher likelihood of depressive symptoms. Previous research also found that higher score of exclusion from civic participation and social relationships negatively affect the overall wellbeing among elderly that may increase depressive symptoms [48].

We also find that non-voting behaviour in the domains of civic participation and social relations is also linked with a higher likelihood of depressive symptoms among the elderly in India. Although Gagné and colleagues (2020) argued that older persons’ voting behaviour may be affected by geriatric symptoms such as different disabilities and other physical and mental health-related concerns [53]. Studies focusing on voting behaviour in the context of social exclusion suggest that regardless of health concerns and disability among the elderly, non-participation in political activity can leave elderly feeling excluded from overall political participation or involvement, resulting in an increase in depressive symptoms among older adults [54, 55].

While, receiving poorer services at stores/restaurants and lack of support to health services as the predominant domain of social exclusion resulting in high depressive symptoms among Indian elderly. These findings are in line with other existing studies [15, 34, 56, 57]. According to research on social exclusion and subjective well-being among older Europeans, the most significant variables affecting life satisfaction, happiness, and overall health are exclusion from financial resources and basic services [17]. While, Chae & Lee (2018) demonstrated that older people are more likely to be depressed when they are denied access to essential services in rural Korea [56]. The most likely reason in this context would be the age related discrimination that the elderly are widely faced merely due to their age. The body of literature on age discrimination highlighted that elderly are often given less important in the public space for instance in market, restaurant, public health care clinics which degrade their perceived health condition, overall wellbeing increasing the depression and anxiety level among older individuals [58, 59]. Existing research indicates that agism may be a potential explanation for the connection between increasing exclusion from services and an increase in depressive symptoms among older individuals. Globalization, urbanisation, and the rise of the nuclear family structure in India have recently posed challenges to the core societal norms and traditions surrounding older individuals. In the setting of India, older adults are typically regarded and treated with great respect. Thus, the fundamental changes might result to an increase in socail exclusion in India, which may worsen depression symptoms in older persons.

While the findings of this study provide a significant original addition to our understanding of social exclusion and depression, particularly in the context of India’s expanding elderly population, the study has a number of limitations. First, Due to a lack of information, only two domains have been included in the current research, despite the fact that several international studies have advised to include a range of domains to define social exclusion, such as cultural exclusion, exclusion from the neighboring community [46–48]. Second, Due to the cross-sectional structure of the dataset, possible causal effects of social exclusion on depression symptoms are absent in this study. Third, it is possible that social exclusion and depressive symptoms have a bidirectional relationship that has not been investigated in this research. Therefore, more research is required to determine the direction of the link between social isolation and depressed symptoms in Indian settings.

## Conclusion

The findings of this study shed light on social exclusion and its connection with depression symptoms among elderly Indians. Our study reveals that the most visible manifestations of social exclusion are in terms of civic participation, relationships, and exclusion from service. Social exclusion also has a link to depressive symptoms, with a greater likelihood among non-voters, those who live alone, and those who are mistreated when seeking services. The study’s significance is that elder health care programs should be broadened in scope while preventing social exclusion, producing significant improvements in mental health in older adults. There is a need for social policy that focuses on community-based care for older individuals, particularly those addressing several aspects of social exclusion.

## Supplementary Information

**Additional file 1**

## Data Availability

The study utilizes a secondary source of data that is freely available in the public domain through https://g2aging.org/.

## Abbreviations

OR: Odds ratio
UOR: Unadjusted odds ratio
AOR: Adjusted odds ratio
CI: Confidence Interval
