# Supplementary material for: Social exclusion and mental health among older adults: cross-sectional evidence from a population-based survey in India
Source: BMC Psychiatry. 2022 Jun 18;22:409. doi: 10.1186/s12888-022-04064-1 (PMC9206346; doi:10.1186/s12888-022-04064-1)
Supplement: Supplementary file 1 — Additional file 1 [file 12888_2022_4064_MOESM1_ESM.docx]

|  | **Model 1** | **Model 2** | **Model 3** | **Model 4** | **Model 5** |
| --- | --- | --- | --- | --- | --- |
|  | **AME in pp (CI at 95%)** | **AME in pp (CI at 95%)** | **AME in pp (CI at 95%)** | **AME in pp (CI at 95%)** | **AME in pp (CI at 95%)** |
| **Social exclusion** |  |  |  |  |  |
| ***Civic activity & social relation exclusion score*** |  |  |  |  |  |
| 0 (ref.) |  |  |  |  |  |
| 1 |  | -0.002 (-0.024  0.02) | -0.013 (-0.036  0.01) |  |  |
| 2 |  | -0.021**(-0.041  -0.001) | -0.041***(-0.062  -0.02) |  |  |
| 3 |  | 0.023**(0.003  0.044) | -0.009 (-0.03  0.012) |  |  |
| 4 |  | 0.13***(0.097  0.163) | 0.066***(0.033  0.1) |  |  |
| ***Service exclusion score*** |  |  |  |  |  |
| 0 (ref.) |  |  |  |  |  |
| 1 |  | 0.236***(0.21  0.262) |  | 0.226***(0.2  0.252) |  |
| 2 |  | 0.351***(0.322  0.381) |  | 0.346***(0.317  0.376) |  |
| ***Overall social exclusion score*** |  |  |  |  |  |
| 0 (ref.) |  |  |  |  |  |
| 1 |  | 0.015 (-0.007  0.037) |  |  | 0.007 (-0.016  0.03) |
| 2 |  | 0.02*(0  0.041) |  |  | 0.005 (-0.017  0.026) |
| 3 |  | 0.072***(0.052  0.093) |  |  | 0.046***(0.025  0.068) |
| 4 |  | 0.211***(0.182  0.24) |  |  | 0.166***(0.136  0.196) |
| 5 |  | 0.339***(0.286  0.391) |  |  | 0.297***(0.244  0.35) |
| 6 |  | 0.307***(0.163  0.451) |  |  | 0.246***(0.102  0.39) |
| **Age group** |  |  |  |  |  |
| 60-69 (ref.) |  |  |  |  |  |
| 70-79 | 0.024***(0.012  0.035) |  | 0.023***(0.012  0.035) | 0.025***(0.013  0.036) | 0.021***(0.01  0.033) |
| 80+ | 0.058***(0.04  0.076) |  | 0.055***(0.037  0.073) | 0.059***(0.042  0.077) | 0.05***(0.032  0.068) |
| **Place of residence** |  |  |  |  |  |
| Rural (ref.) |  |  |  |  |  |
| Urban | -0.015***(-0.026  -0.004) |  | -0.016***(-0.027  -0.005) | -0.016***(-0.027  -0.005) | -0.016***(-0.027  -0.005) |
| **Gender** |  |  |  |  |  |
| Men (ref.) |  |  |  |  |  |
| Women | 0.012**(0.001  0.024) |  | 0.013**(0.002  0.025) | 0.017***(0.005  0.028) | 0.01*(-0.001  0.022) |
| **Marital status** |  |  |  |  |  |
| Married (ref.) |  |  |  |  |  |
| Non-married | 0.055***(0.043  0.066) |  | 0.047***(0.035  0.059) | 0.052***(0.04  0.064) | 0.039***(0.027  0.051) |
| **Educational status** |  |  |  |  |  |
| No formal education (ref.) |  |  |  |  |  |
| Formal education | -0.045***(-0.056  -0.033) |  | -0.043***(-0.055  -0.032) | -0.035***(-0.046  -0.024) | -0.036***(-0.047  -0.025) |
| **Wealth index** |  |  |  |  |  |
| Poor (ref.) |  |  |  |  |  |
| Middle | -0.034***(-0.047  -0.02) |  | -0.034***(-0.048  -0.021) | -0.033***(-0.047  -0.02) | -0.033***(-0.046  -0.019) |
| Rich | -0.024***(-0.035  -0.012) |  | -0.026***(-0.037  -0.014) | -0.025***(-0.036  -0.014) | -0.025***(-0.036  -0.013) |
| **Note:** AME denotes Averaged marginal effects; pp denotes percentage points. Estimated averaged marginal effects on probability of the depressive symptoms from logistic regressions | | | | | |

**Appendix Table 1:** Averaged marginal effects on probability of depressive symptoms among older adults aged 60 years and above in India, LASI wave 1, 2017-18 (N=31,464).
